# Observations on Reform

**Published:** 1810-02

**Authors:** 


*January* 11, 1810.

---

### *To the Editors of the Medical and Physical Journal.*

Gentlemen,

As the Medical Journal does not reach me till the month in which it is published is far advanced, and as the season is become rather more unhealthy, other engagements press upon my notice, I have not leisure at present to resume the subject of my former communication in the manner I could wish, and must content myself with a hasty reply to the observations made in your last Number, in so far as I find myself immediately affected by them.

I may premise, that I am by no means an enemy to a plan of general and gradual Reform; to use a figure which has of late been much usurped by political writers, such a course of alteratives as will effect a radical change in the body-professional, and render the streams more clear by purifying and improving their source. But in calling myself an enemy to the quackery of corporate bodies, which your your liberal and well informed Correspondent thinks is quaintly expressing nothing at all, I mean that kind of quackery which prescribes certain and absolute rules, and precise enactments for remedying certain evils, some of which, if they really exist, are to be referred for their causes to radical defects, out of the reach of legal nostrums. To exemplify my meaning, and that the drift of my argument may be better understood, I would ask your Correspondent, to what we may attribute the great increase of empiricism? Not, I should presume, to want of numbers, nor the want of skill, in the regular practitioners. This subject I know has been amply discussed; but of the causes assigned for this increase, I know of none more weighty than the increasing opulence and luxury of certain ranks of men; the consequent leisure, pusillanimity, and credulity with regard to medical subjects, which are naturally induced in such situations; and the pernicious example of benevolent and well-meaning, but weak and ill-directed people, the Buchans and Bountifuls, who infest the land with their cant, and their small-ware-acts of generosity.

I did not require Dr. Harrison to publish the whole bill, which it is intended to lay before parliament, but to give those who have not had the means of understanding thoroughly what was meant to be proposed, some better information of what they were called upon to pay for; and as your Correspondent asserts, that our gains are few, he will not wonder that we are inclined to ask, *cui bono*, before we open our purses. I am not versed in parliamentary matters, but I have no notion of a thing being unparliamentary, of which that "August Assembly," as yet, has taken no cognizance. The explanation which your Correspondent has given, has certainly obviated some of my objections to the measure; yet still I fear that, at best, it will not answer the expectations of its advisers.

I shall not stop to trace how the present pre-eminence of the law over other professions has arisen, and that we are at present blessed with lawyer rule; if I were, I think it could be proved that such pre-eminence has rather been the cause than the effect of those ordinances, by which your Correspondent thinks its professors have been so much benefitted. The multiplication of laws, the multiplicity of taxes, and the division of property of a mercantile country, all explain why the profession of the law should be more lucrative, and more commanding; and since the faculty of talking is become the road to power, it is not surprising that lawyers should usurp it. But I still maintain tain, that in so far as my observation extends, the profession of physic has lost no ground in point of respectability, except perhaps within a certain London circle, where riches have gained a temporary superiority over information and intellect; and that in the part of the country where I reside, my Lady gives her hand at a ball to her medical attendant, with as much respect, and as much freedom, as she does in the sick chamber. As to the remarks of Viator, I presume that the rule of precedence, which he has given upon the authority of the Herald's Office, holds only within the naval and military establishments, where every thing ranks subordinate to the profession of arms; and on board the fleet, I believe, an admiral takes place of an earl. But in society at large, and in promiscuous assemblies, such a degradation of either of the learned faculties is far from being recognised; and in common language, the professions of law, physic, and divinity, still hold an equal rank. If such is the courtesy of the country, as stated by the Herald's Office, doubtless it is given upon the most ancient authority, and is an additional proof of the amelioration of the condition of our profession, which now holds a rank, in common acceptation, far above that it held some centuries ago. The assertion of your Correspondent, that medical men in the country, are the associates of the steward's room, and the butt of the 'squire, scarcely merit notice; for it is, as far as I am acquainted, perfectly unfounded, and is what does not exist, except in some novels and plays, amongst which, ridicule of the learned professions is handed about as a standing dish. Your Correspondent seems also to doubt the superior estimation in which medicine is held in this island. I remember a friend of mine, who had travelled upon the continent, once told me, that he always found it more to his advantage to pass for a simple *savant*, than to exercise the right of an M. D. or to be esteemed a physician.

In a moral and political point of view, the ascendancy which the science of medicine and its professors have over the minds of the present enervate generation, is sufficient for its purpose; it is too great, if we may credit the elegant Rosseau. "Cet art mensonger plus fait pour les maux de l' esprit que pour ceux des corps, n'est pas plus utile aux uns qu'aux autres; il nous guérit moins de nos maladies qu'il ne nous en emprime l'effroi. Il recule moins la mort qu'il ne la fait sentir d'avance; il use la vie au lieu de la prolonger. Voulez-vous, trouver des hommes d'un vrai courage? Cherchez-les dans les lieux où il n'y a point point de medecins, ou l'on ignore les consequences des maladies, et ou l'on ne songe guère à la mort." The immense numbers, particularly of the female sex, who live the slaves of their physicians, and the victims of unfounded apprehensions of disease, almost warrant the conclusion of this strict observer of human nature. I have been led into this digression by the comparison drawn between the relative situation of law and physic. If the former has taken too much hold upon our property and our actions, may not the latter usurp too great an authority over our minds?

To a well digested plan of reform, directed chiefly against empiricism, I have no objection; and it may be extended towards the improvement of the medical school, to the exclusion of ignorance and incompetence there; but for the rest of the profession, I should wish to see it make its own way, and find its own level.

If Dr. Harrison, or any of the projectors of the bill, will assure us of their having provided against the invasion of corruption amongst those from whom authority to practice is to be obtained, and of their lasting competence to the task of judging: If they will assure us that the caprice of individuals, and the caprice of men, will not render restriction nugatory; and that in the lapse of a few years, it will not be found that their laws are binding only upon those who have entered within their immediate limits: I am ready to subscribe my guinea towards the expence of an Act of Parliament; and if we are indeed sunk so low, as not to class with the children of the sister arts, I am willing to concur in any measure which will convince the world, that we still aspire to the situations formerly held by Locke, Garth, and Ratcliffe, and their contemporaries.

*January* 14, 1810.

